# IMPACT OF HELICOBACTER PYLORI ON EARLY POSTOPERATIVE COMPLICATIONS AFTER SLEEVE GASTRECTOMY: A SYSTEMATIC REVIEW AND META-ANALYSIS

**DOI:** 10.1590/0102-672020230070e1788

**Published:** 2024-02-02

**Authors:** Anna Carolina Batista DANTAS, Vitoria Ramos JAYME, Kaique Flavio Xavier Cardoso FILARDI, Denis PAJECKI, Marco Aurelio SANTO

**Affiliations:** 1 Universidade de São Paulo, Faculty of Medicine, Department of Gastroenterology, Bariatric and Metabolic Surgical Unit – São Paulo (SP), Brazil.; 2 Universidade de São Paulo, Faculty of Medicine, Department of Gastroenterology – São Paulo (SP), Brazil.

**Keywords:** Bariatric Surgery, Helicobacter Pylori, Postoperative Complications, Cirurgia Bariátrica, Helicobacter Pylori, Complicações Pós-Operatórias

## Abstract

The impact of *Helicobacter pylori* (HP) on postoperative outcomes after sleeve gastrectomy (SG) is still controversial. A systematic review and meta-analysis were performed to compare the incidence of early complications after SG between HP-positive and HP-negative patients. Eight retrospective comparative studies were included, comprising 4,877 individuals. The prevalence of HP infection in gastric resected specimens ranged from 7.77 to 43.20%. There were no statistically significant differences between groups for overall complications (OR 1.46; 95%CI 0.95–2.23; p=0.08), bleeding (OR 1.35; 95%CI 0.70–2.60; p=0.38), and leak (OR 1.74; 95%CI 0.80–3.81; p=0.17) rates. The need for routine screening and treatment of HP infection before SG remains ambiguous.

## RESUMO

O impacto do *Helicobacter pylori* (HP) nos desfechos pós-operatórios da Gastrectomia Vertical (GV) ainda é controverso. Uma revisão sistemática e meta-análise foram realizadas para comparar a incidência de complicações pós-operatórias depois da GV entre pacientes HP-positivo e HP-negativo. Oito estudos comparativos retrospectivos foram incluídos, englobando 4.877 indivíduos. A prevalência de infecção por *HP*nos espécimes gástricos ressecados variou de 7,77 a 43,20%. Não houve diferença estatística significante entre os grupos para complicações gerais (OR 1,46; IC95% 0,95–2,23; p=0,08), sangramento (OR 1,35; IC95% 0,70–2,60; p=0,38), e fístula (OR 1,74; IC95% 0,80–3,81; p=0,17). A necessidade de rastreio e erradicação rotineira do HP antes da GV permanece ambígua.

## INTRODUCTION

H*elicobacter pylori* (HP) infection is one of the most common worldwide, prevalent in almost half the global population^15^. Despite being asymptomatic in over 80% of cases^4^, it is associated with gastritis, peptic ulcer disease, and non-cardia gastric adenocarcinoma^15,21^.

Evidence shows that HP infection is more prevalent in patients with obesity than in the general population^3,10^. This leads to the adoption of preoperative routine HP screening and eradication in some metabolic and bariatric surgery (MBS) centers^12,27^, albeit strong evidence of routine HP eradication in this setting is limited.

The impact of HP on perioperative outcomes of MBS is still controversial. In a nationwide analysis with 253,765 patients, HP was the strongest independent predictor of marginal ulceration^25^. In contrast, other studies found no association between HP infection and surgical complications^16,19^. While these data focus on anastomotic complications related to Roux-en-Y gastric bypass (RYGB), the influence of HP infection on sleeve gastrectomy (SG), the most performed bariatric procedure nowadays, is still limited to small retrospective studies^19,31^.

The present study aimed to perform a systematic review and meta-analysis to determine the impact of HP on early postoperative complications after SG.

## METHODS

### Literature search

A systematic search was performed in Embase and PubMed databases, and grey literature, from inception to January 28, 2023, based on the Preferred Reporting Items for Systematic Reviews and Meta-Analysis (PRISMA) statement^20^. The search strategy for PubMed was (sleeve gastrectomy) AND (complication) AND ((helicobacter pylori) OR (h pylori)). A similar search strategy was used for other databases. The study was registered in the International Prospective Register of Systematic Reviews (PROSPERO) - University of York with Registry Number CRD42022385224.

### Study selection

Two reviewers (ACBD and VRJ) independently screened all potentially relevant titles and abstracts for eligibility. Abstracts were screened based on the following inclusion criteria: patients undergoing SG, with documented HP infection on resected pathologic specimen, and reporting on perioperative outcomes. Discrepancies were solved by consensus or through assessment by a third independent reviewer (KFXCF). Studies with preoperative eradication of HP or without comparative groups between positive and negative HP were excluded, in addition to editorials, letters, conference proceedings, reviews, case reports, and animal models. No restrictions were set for language.

### Data extraction

Study details, patient characteristics, and outcome data were independently extracted by two reviewers (ACBD and KFXCF). Our primary outcome was overall early postoperative complications. Secondary outcomes were bleeding and leak.

The following data were obtained from the included studies: first author, journal, year of publication, study design, number of patients in each group, age, gender, and preoperative body mass index (BMI).

### Quality assessment

This meta-analysis was conducted according to the consensus statement for meta-analysis of observational studies (MOOSE)^30^. Two reviewers (VRJ and KFXCF) independently assessed the quality of studies using the modified Newcastle–Ottawa Scale (NOS) quality assessment tool for observational studies^32^. Possible conflicts were discussed with a third reviewer (ACBD).

### Statistical analysis

Outcomes assessed in this meta-analysis included overall complications, leak, and bleeding rates. The estimated effects were calculated using ReviewManager (RevMan) 5 software from the Cochrane website^22^. The odds ratio (OR) was calculated with the respective 95% confidence interval (95%CI). The statistical heterogeneity was assessed with the I-squared (*I* ^2^) statistic. A random effects model (restricted maximum likelihood) was applied due to the significant clinical heterogeneity among the included studies. Statistical significance was set at p=0.05.

## RESULTS

### Study selection

**Figure 1:**
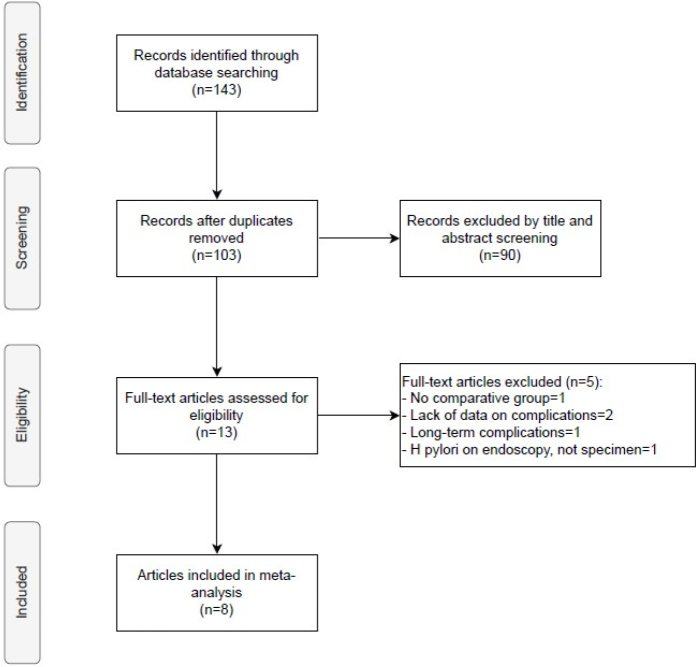
Flow diagram of study selection.

Through a preliminary database search, 143 studies were obtained. After removing duplicates, 103 titles and abstracts were screened, leaving 13 studies for full-text evaluation. As shown in the flowchart (Figure 1), only eight manuscripts fulfilled the eligibility criteria of this meta-analysis, all retrospective comparative analyses.

### Study characteristics

A total of eight studies were included, comprising 4,877 individuals^1,2,6,8,13,23,26,28^. The main characteristics and quality assessment of all included studies are shown in Table 1. Preoperative upper gastrointestinal endoscopy (UGE) and HP testing were heterogeneous among studies: Emile et al.^8^ and Abu-Abeid et al.^1^ did it routinely, Almazeedi et al.^2^ at the surgeon’s discretion, and all the others^6,13,23,26,28^ did no HP screening before surgery. If HP was positive on gastric pathology specimens, only half of the studies treated it with outpatient oral antibiotics^2,8,13,28^. Most of the studies considered early complications 30 days after the procedure^1,6,13,23,26,28^, except two that had no mention of follow-up period for perioperative morbidity^2,8^.

**Table 1:** Baseline characteristics of included studies.

Author	Year	Study design	Quality assessment*	n	Study arm	Patients (n)	Mean age years	Gender %female	Mean BMI kg/m^2^
Almazeedi et al.^2^	2014	Retrospective	7	682	HP positive HP negative	53 629	33.5 33.5	- -	46.0 46.0
Rossetti et al.^23^	2014	Retrospective	8	184	HP positive HP negative	72 112	32.4 37.3	70.83 60.71	44.4 47.2
Brownlee et al.^6^	2015	Retrospective	8	490	HP positive HP negative	52 438	40.7 40.2	80.77 70.09	48.2 47.0
Gonzalez-Heredia et al.^13^	2015	Retrospective	8	400	HP positive HP negative	68 332	42.2 41.3	86.76 81.63	51.5 50.6
Shanti et al.^28^	2017	Retrospective	8	500	HP positive HP negative	216 284	33.3 36.0	71.76 72.89	46.2 45.0
Serin et al.^26^	2018	Retrospective	8	460	HP positive HP negative	150 310	38.4 37.1	66.00 73.23	42.9 42.6
Emile et al.^8^	2020	Retrospective	8	176	HP positive HP negative	69 107	33.9 34.4	88.41 85.98	50.8 48.8
Abu-Abeid et al.^1^	2022	Retrospective	8	1,985	HP positive HP negative	179 1806	40.4 41.7	63.13 65.84	43.4 42.6

* Newcastle-Ottawa Scale assessment of cohort studies, maximum score of 9. BMI: body mass index; HP: *Helicobacter pylori*.

### Outcomes

The prevalence of HP infection ranged from 7.77 to 43.20%. In those with HP positive, the mean age was 36.9 years old, the mean BMI was 46.7 kg/m^2^, and 75.38% were female. The following complication rates were observed in this group throughout the studies: overall complications 0.00–10.06%, bleeding 0.00–3.91%, and leak 0.00–2.90%. Of those HP-negative patients, the mean age was 37.7 years old, the mean BMI was 46.2 kg/m^2^, and 72.91% were female. Complication rates among the studies were: overall complications 0.30–8.53 %, bleeding 0.00–2.71 %, and leak 0.00–2.68%.

### Meta-analysis

**Figure 2:**
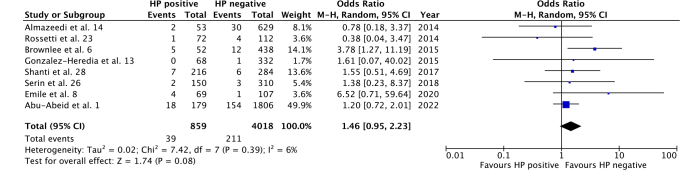
Overall complication rates for HP-positive vs. HP-negative groups.

**Figure 3:**
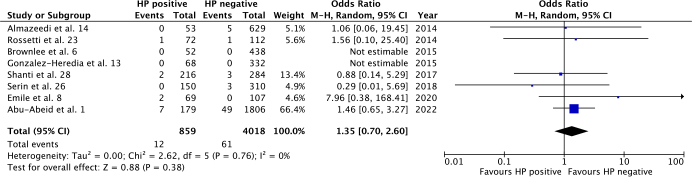
Bleeding rates for HP-positive vs. HP-negative groups.

HP: *Helicobacter pylori*; CI: confidence interval; M-H: Mantel-Haenszel statistics.

**Figure 4:**
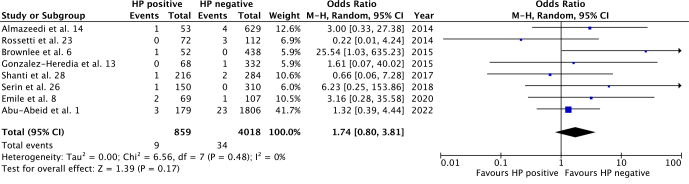
Leak rates for HP-positive vs. HP-negative groups.

HP: *Helicobacter pylori; CI: confidence interval; M-H: Mantel-Haenszel statistics.*

All studies reported overall complications, bleeding, and leak rates, with sufficient data for a meta-analysis. Overall complications had no significant difference between HP positive and negative groups (OR 1.46; 95%CI 0.95–2.23; p=0.08), as seen in Figure 2. Accordingly, there was no statistically significant difference between groups for bleeding (OR 1.35; 95%CI 0.70–2.60; p=0.38) and leak (OR 1.74; 95%CI 0.80–3.81; p=0.17) rates (Figures 3 and 4). Heterogeneity was not significant for all outcomes (p>0.05).

HP: *Helicobacter pylori;* CI: confidence interval; M-H:Mantel-Haenszel statistics.

## DISCUSSION

This systematic review and meta-analysis summarized and compared early postoperative complications after SG according to HP infection status on gastric pathology specimens. There were no statistically significant differences between groups for overall complications, bleeding, and leak.

Some studies showed high rates (>30%) of HP infection, but there was no correlation with preoperative screening^8,23,26,28^. Abu-Abeid et al.^1^ were the only ones to do routine preoperative screening and treatment for HP; with all HP negative on preoperative urea breath test, still, one-third (34.55%) were positive on resected specimens. HP eradication, however, had no impact on overall and major complications.

Although two studies showed higher complication rates for patients who were positive for HP^8,28^, none of the studies included in this meta-analysis demonstrated a significant impact of HP infection on postoperative complications after SG. The study by Emile et al.^8^ was the only to observe a higher incidence of overall and specific complications in the HP-positive group, but also not statistically significant. Ergin et al.^9^ evaluated the impact of preoperative HP eradication on gastric wall thickness and found that antrum mucosal thickness was significantly higher in the HP-positive group without eradication treatment, but this did not affect early postoperative complications.

A previous meta-analysis evaluated the impact of HP on postoperative outcomes after bariatric surgery^19^. While it included a range of MBS procedures, such as SG, RYGB, and vertical banded gastroplasty (VBG), it was also heterogeneous in diagnostic criteria for HP infection and follow-up time, including early and long-term morbidity. In addition, HP was only associated with increased marginal ulceration rates, but this finding could not be meta-analyzed as it was based on a lone populational study with more than 250,000 individuals^25^.

Beyond the risk of perioperative complications, the impact of HP on other outcomes has been increasingly explored in the literature. There is growing evidence for a potential association between HP infection and metabolic syndrome^17^. Goday et al.^11^ evaluated if HP eradication treatment could influence the evolution of weight loss and metabolic markers after bariatric surgery and found that HP-treated patients had higher weight loss and higher reduction of triglycerides and glucose levels. Moreover, the impact of HP has been evaluated on the incidence of gallstone formation after SG and found anti-HP antibody seropositive to be associated with cholelithiasis^14,29^. Its relation to gastroesophageal reflux disease (GERD) has also been explored, but despite no association with the incidence of *de novo* GERD, improvement of symptoms was higher in those with HP-confirmed eradication than in those without HP infection.

There is still no consensus on the role of routine preoperative screening and eradication of HP before bariatric surgery^12^. The International Federation for the Surgery of Obesity and Metabolic Disorders (IFSO) position statement recommends routine UGE on candidates for MBS^5^, and HP infection is one of the most common abnormal findings on preoperative UGE^24^. Based on the recent update of the Maastricht VI/Florence consensus report recommending that all patients with evidence of active infection with HP should be offered treatment^18^, and considering a recent international survey showing that two-thirds of experts (69%) already do it before MBS^7^, it may be time for us to routinely screen and eradicate HP in this population, despite no impact on early postoperative complications as found in this meta-analysis.

This systematic review is not without its limitations. Only eight retrospective studies met our inclusion criteria and were limited by the small sample size and its retrospective nature. In addition, all studies already had the same finding that HP infection was not significant for early perioperative complications after SG, thus bringing no new conclusions from this meta-analysis. Despite these limitations, this review is novel in its updated analysis of the literature focused solely on SG.

## CONCLUSIONS

The presence of *Helicobacter pylori* on the resected gastric specimen after sleeve gastrectomy was not found to be adversely associated with early overall postoperative complications, bleeding, or leak. There is, however, no recommendation against routine screening and treatment of HP infection before metabolic and bariatric surgery, as other outcomes may be involved.
